# Prototyping Trastuzumab Docetaxel Immunoliposomes with a New FCM-Based Method to Quantify Optimal Antibody Density on Nanoparticles

**DOI:** 10.1038/s41598-020-60856-z

**Published:** 2020-03-05

**Authors:** A. Rodallec, C. Franco, S. Robert, G. Sicard, S. Giacometti, B. Lacarelle, F. Bouquet, A. Savina, R. Lacroix, F. Dignat-George, J. Ciccolini, P. Poncelet, R. Fanciullino

**Affiliations:** 1 SMARTc Unit, CRCM, Inserm UMR1068, CNRS UMR7258, Aix-Marseille University, Marseille, France; 2 Biocytex, Marseille, France; 3 C2VN, AMUTICYT Core facility, INSERM, INRA, Aix-Marseille University, Marseille, France; 4 Aix-Marseille University, INSERM, INRA, C2VN UMR_S1263, UFR de Pharmacie, Marseille, France; 5 Institut Roche, Boulogne Billancourt, France; 6 Department of Hematology and Vascular Biology, CHU La Conception, APHM, Marseille, France

**Keywords:** Nanotechnology in cancer, Nanobiotechnology, Flow cytometry

## Abstract

Developing targeted nanoparticles is a rising strategy to improve drug delivery in oncology. Antibodies are the most commonly used targeting agents. However, determination of their optimal number at the surface remains a challenging issue, mainly due to the difficulties in measuring precisely surface coating levels when prototyping nanoparticles. We developed an original quantitative assay to measure the exact number of coated antibodies per nanoparticle. Using flow cytometry optimized for submicron particle analysis and beads covered with known amounts of human IgG-kappa mimicking various amounts of antibodies, this new method was tested as part of the prototyping of docetaxel liposomes coated with trastuzumab against Her2+ breast cancer. This quantification method allowed to discriminate various batches of immunoliposomes depending on their trastuzumab density on nanoparticle surface (i.e., 330 (Immunoliposome-1), 480 (Immunoliposome-2) and 690 (Immunoliposome-3), p = 0.004, One-way ANOVA). Here we showed that optimal number of grafted antibodies on nanoparticles should be finely tuned and highest density of targeting agent is not necessarily associated with highest efficacy. Overall, this new method should help to better prototype third generation nanoparticles.

## Introduction

Development of nanoparticles (NP) for targeted delivery and controlled drug release may improve the therapeutic index of drugs, especially that of anticancer agents. Such improvement is particularly relevant when administering cytotoxics that show frequently dose-limiting toxicities, thus resulting in suboptimal efficacy^1^. In addition to passive targeting through the Enhanced Permeation and Retention effect^2,3^, active targeting is based upon receptor-mediated binding of nanoparticles to the membrane of tumor cells. A successful and actively targeted nanomedicine requires therefore a fine balance of ligand content and surface exposure of cell-binding moieties that minimize immunological recognition and rapid clearance by macrophages and scavenging cells. The current methods for post-synthesis NP surface modification often require multi-steps complicated procedures, thus making difficult to achieve batch-to-batch reproducibility^4–6^. This calls for the need to develop post-synthesis analytical methods to control the surface properties of the NP, such as the exact coating rate when a monoclonal antibody is to be used as a targeting agent. To date, no such method has been made available and little information is usually provided regarding the exact number of monoclonal antibodies coated on third generation nanoparticles such as immunoliposomes. Assaying monoclonal antibodies in pharmaceutical preparations is mostly based upon Enzyme Linked ImmunoSorbent Assay (ELISA) techniques. Indirect methods such as Bradford assay or Pierce BCA protein assay have been proposed, but they can only provide semi-quantitative information and are in no way suitable to precisely measure the exact amount of antibodies grafted on a nanoparticle surface^7–10^. Flow Cytometry (FCM) is one of the rare rapid, multi-parametric technologies that offers single particle analysis. This technique has already demonstrated its high interest in the characterization of both biological and synthetic particles^11,12^. Interestingly, such FCM-based quantitative analysis of immuno-staining should be applied on an absolute rather than only relative basis through *ad hoc* calibration^13^. This allows reproducible measurements at various time points providing more meaningful results expressed as molecules/cell rather than in arbitrary units (a.u.) of fluorescence^13–18^. This “quantitative FCM” (QFCM) approach, allowing absolute quantification of membrane antigens, has already found several experimental or clinical applications^19^. With the rise of biotherapies, such quantitative approach could be helpful in biopharmaceutical development^20–22^ both to measure the expression of target antigens and to characterize new entities^23,24^. The aims of this work were first to develop an original QFCM assay to measure the number of antibodies coated on the surface of submicron-sized particles and second to evaluate the potential impact of antibodies coating level on immunoliposomes cytotoxic effect.

## Results

### FCM method: development of a quantification assay for submicron particles

**Figure 1:**
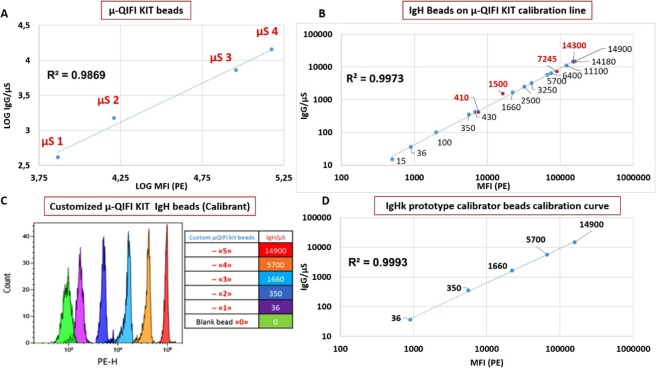
Aspect and calibration of the prototyped “IgHk calibrator beads”. (**A**) Calibration line of mouse IgG coated µ-QIFIkit calibrator beads on CyTOFLEX S (Beckman coulter, Villepinte France) for the calibration of human IgG kappa light chains coated beads (IgHk). (**B**) Quantification of Human IgHk on all prototyped IgHk calibrated beads using µ-QIFIkit as reference. IgHk beads have been first saturated with unlabeled L1C1 Mab, washed and fluorescently stained with PE-labeled anti-mouse IgG sencondary reagent in parallel with the µ-QIFIkit beads used as reference. (**C**) Superposition of histograms from the prototyped “IgHk calibrator beads” as seen on the PE channel of CytoFLEX S under yellow laser excitation. A limited series of 5 beads regularly distributed over the measuring range were chosen from the larger series of all (n = 13) calibrated beads to be later used in direct IF experiments with L1C1-PE as “IgHk calibrator beads”. (**D**) Calibration line of the prototyped “IgHk calibrator beads” generated for this study.

As described in detail in the Supplemental data section, the 10 µm QIFIkit calibrator, was scaled-down to generate a series of prototype 1 µm-sized “µ-QIFIkit” calibrator beads covering an approximate range of ~20 to ~20,000 mouse IgG/bead. The correlation coefficient r² of the prototype was equal to 0.9869 (Fig. 1A).

For further quantitation experiments on liposomes, a smaller series of 5 regularly spanned bead subsets were selected (Fig. 1B,C) to create a prototype “IgHk calibrator kit”, i.e.: 36-350-1,660-5,700-14,900 IgHk molecules/bead, considered equivalent to 18-175-830-2,850-7,450 human IgG molecules/bead, based on the theoretical expression of two kappa light chains per IgG. The correlation coefficient of this new IgHk prototype was 0.9993 (Fig. 1D).

### Detection of immunoliposomes and quantification of coated trastuzumab on FCM

**Figure 2:**
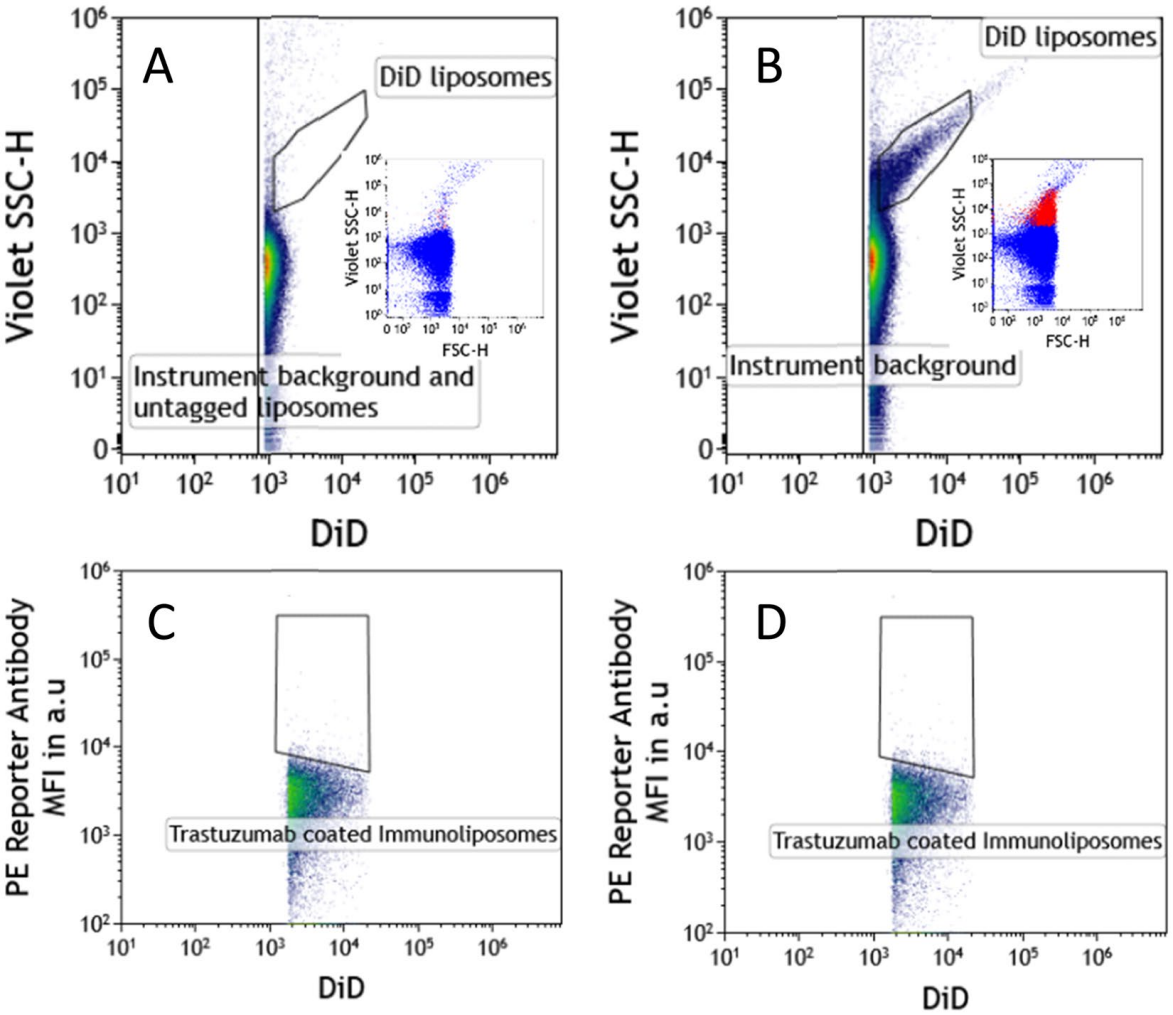
FCM analysis of non-fluorescent immunoliposomes and instrument background noise (**A**), DiD+ immunoliposomes (**B**), DiD+ Trastuzumab uncoated liposomes (**C**) and DiD+ Trastuzumab coated immunoliposomes (**D**). Inset figures present FSC-SSC plots showing the distribution of background noise events in blue dots (insets A and B) and specific detection of DID stained liposomes with red dots (inset B).

**Figure 3:**
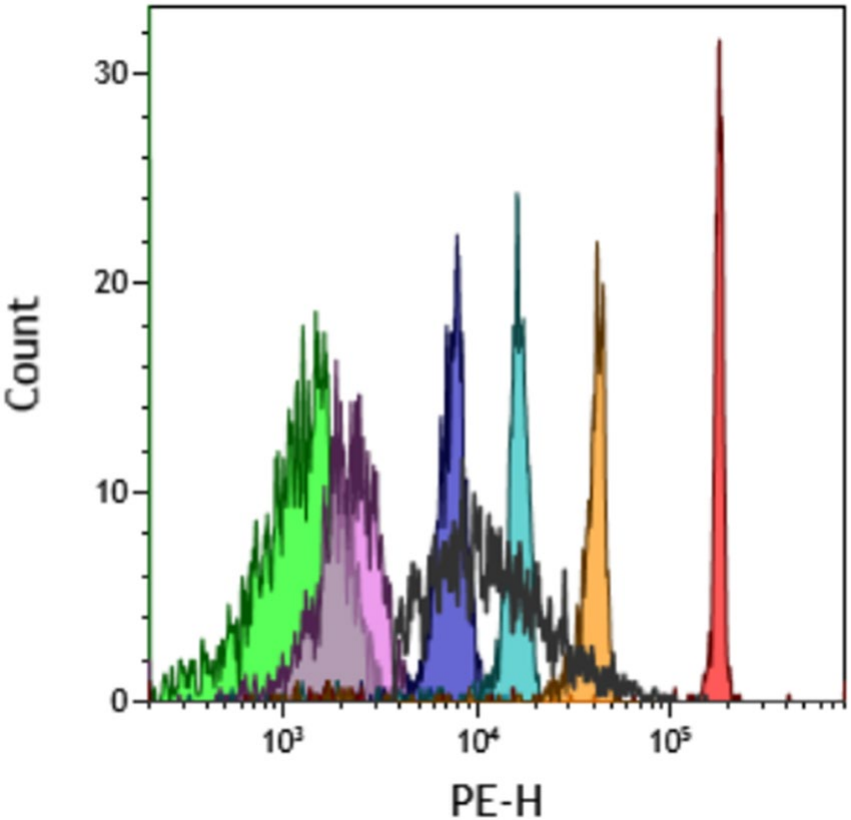
Overlay plot in the revelation Antibody PE fluorescence channel. Color lines: IgGH calibrator beads coated with 0 (green), 18 (pink), 175 (violet), 830 (blue), 2850 (orange) and 7450 (red) IgGH antibodies respectively. Black line represents PE distribution of trastuzumab coated DiD+ immunoliposomes.

As explained in the method section, the triggering parameter for FCM analysis of liposomes was based on DiD fluorescence. The positivity threshold was defined using unstained immunoliposomes (Fig. 2A). Except from electronic and fluidic background, no DiD+ elements were detected. Using tagged liposomes, the SSC/DiD dot plot allowed the definition of the DiD+ (immuno)liposome gate. DiD bright elements were also excluded from the gate due to the possible generation of doublets or multiplets of immunoliposomes. DiD immunoliposomes were successfully detected (Fig. 2B). DiD+ Immunoliposome numeration provided using FCM represented 65% (see Supplemental data section for qNano: measurement of liposome concentration) of total Immunoliposomes counted using TRPS technology (qNano). Moreover, considering the PE quantification channel, the positivity threshold was adjusted using DiD+ uncoated liposomes incubated with the same anti-Human IgG-k PE MAb conjugate (Fig. 2C). This strategy allowed delineating the PE+/DID+ immunoliposome gate on a PE+/DiD+ dot plot (Fig. 2D). Finally, the Median Fluorescence intensity (MFI) of the pre-selected population was collected as arbitrary units of PE fluorescence and converted into the absolute number of kappa light chain/immunoliposome using our IgHk calibrator beads (Fig. 3). Based on the expression of two kappa light chains per IgG, we considered that immunoliposomes may theoretically bind 2 molecules of PE anti-Human IgG reagent (i.e., anti-human kappa light chain monoclonal antibody), resulting in dividing by 2 each result.

Thus, Immunoliposome-1, Immunoliposome-2 and Immunoliposome-3 exhibited 330 ± 30, 480 ± 110 and 690 ± 80 (p < 0.004, One-way ANOVA) coated trastuzumab per liposome, respectively.

### Immunoliposome characterization

No significant difference in size, PDI and entrapment efficiency was observed between immunoliposome batches. According to DLS analysis, immunoliposome population was unimodal in size (i.e., PDI = 0.1 ± 0.01) with a mean diameter of 140 ± 3.4 nm. Docetaxel entrapment efficiency was >90%.

### Stability studies

**Figure 4:**
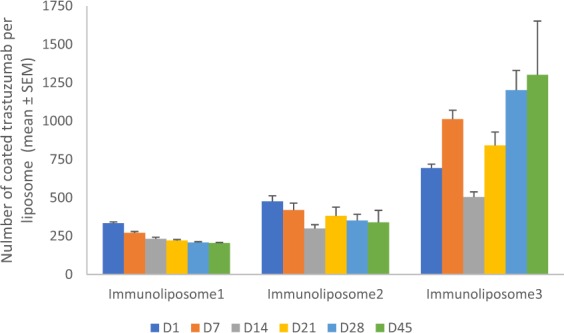
Number of coated trastuzumab on three immunoliposome batches over time^a^. ^a^Values are mean ± SEM of three or more experiments.

No significant difference in stability was observed between immunoliposome batches. As previously published^25^, after 45 days, immunoliposome was steady, PDI increase was not significant (i.e., from 0.1 to 0.153 in 45 days) and mean docetaxel leakage was of 17 ± 13% per week. Evolution in time of coated trastuzumab is illustrated in Fig. 4. After an initial loss of maximum 20% in the first 2 weeks (i.e., 19% and 12%, p > 0.05, *t*-student) for Immunoliposome-1 and Immunoliposome-2, respectively, trastuzumab density remained stable for 45 days (p < 0.05, *t*-student). On the contrary, Immunoliposome-3 trastuzumab density presented strong variations with an apparent increase of 87% at day 45 (p = 0.011, One-way ANOVA).

### *In vitro* efficacy studies: bidimensional (2D) model

**Figure 5:**
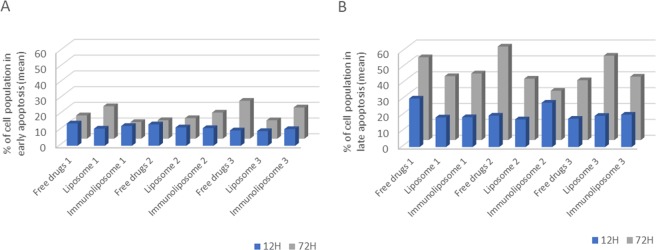
Monitoring of cell population (%) in early (**A**) and late (**B**) apoptosis when treated with three batches of free drugs, liposomes and immunoliposomes for 12 and 72 hours^a^. ^a^Values are mean of three or more experiments.

Empty liposomes and trastuzumab alone did not show any apoptotic effect (data not shown)^7^. For all conditions, no significant difference (p > 0.05, One-way ANOVA) in apoptosis induction was observed between trastuzumab concentrations whether it was free or coated (Supplementary Figs. 2 and 3). For all treatment groups, induction of early apoptosis increased with time (p = 0.004, *t*-test) but no difference between them was observed (Table 1 and Fig. 5A). At 12 and 72 hours, when treated with free docetaxel + trastuzumab, 12 ± 2% and 17 ± 6% of cells were in early apoptosis. Results were 11 ± 1% and 15 ± 5% when treated with liposome + free trastuzumab and 11 ± 1% and 16 ± 5% when treated with immunoliposomes (p = 0.427, One-way ANOVA and p = 0.917, One-way ANOVA, respectively). Similarly, induction of late apoptosis increased with time (p = 0.004, *t*-test) and no difference between treatment groups was observed either (Table 1 and Fig. 5B). At 12 and 72 hours, when treated with free docetaxel + trastuzumab, 23 ± 7% and 50 ± 11% of cells were in late apoptosis. It was 19 ± 1% and 44 ± 8% when treated with liposome + free trastuzumab and 22 ± 5% and 38 ± 11% when treated with immunoliposomes (p = 0.551, One-way ANOVA and p = 0.298, One-way ANOVA, respectively).

**Table 1:** Summary table of cell population (mean % ± SD) in early and late apoptosis when treated with three batches of free drugs, liposomes and immunoliposomes for 12 and 72 hours^a^.

	Early apoptosis 12 H	Early apoptosis 72 H	Late apoptosis 12 H	Late apoptosis 72 H
Free drugs 1	14.2 ± 12.7	14.8 ± 9.3	30.5 ± 5.5	52.1 ±10.1
Liposomes 1	10.8 ± 11.5	20.6 ± 14.7	18.6 ± 8.2	40.3 ± 10.0
Immunoliposome 1	12.5 ± 12.7	10.6 ± 2.8	18.8 ± 6.5	42.0 ± 4.3
Free drugs 2	13.6 ± 15.4	11.7 ± 7.1	19.7 ± 1.3	58.9 ± 3.6
Liposomes 2	11.6 ± 9.4	13.1 ± 10.0	17.4 ± 3.4	38.7 ± 5.0
Immunoliposome 2	11.2 ± 9.2	16.6 ± 13.5	27.9 ± 7.2	31.1 ± 9.7
Free drugs 3	9.7 ± 10.5	24.1 ± 12.2	17.8 ± 2.3	37.7 ± 2.8
Liposomes 3	9.3 ± 9.7	11.7 ± 8.1	19.6 ± 5. 1	53.1 ± 8.1
Immunoliposome 3	10.5 ± 14.2	19.8 ± 10.6	20.3 ± 0.8	39.9 ± 2.6

^a^Values are mean of three or more experiments.

### *In vitro* efficacy studies: three-dimensional (3D) model

**Figure 6:**
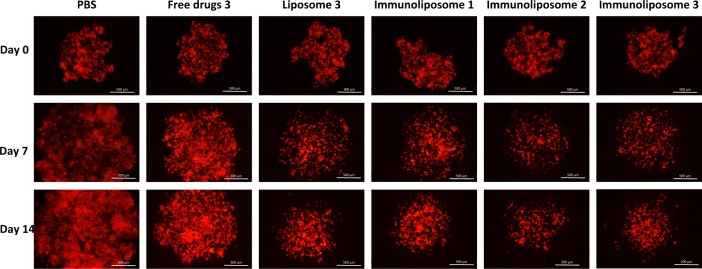
Monitoring of dtomato fluorescent MDA-MB-453 when seeded in 4000 cell spheroids and treated at day 3 and day 10^a^. ^a^Experiment was in triplicate.

**Figure 7:**
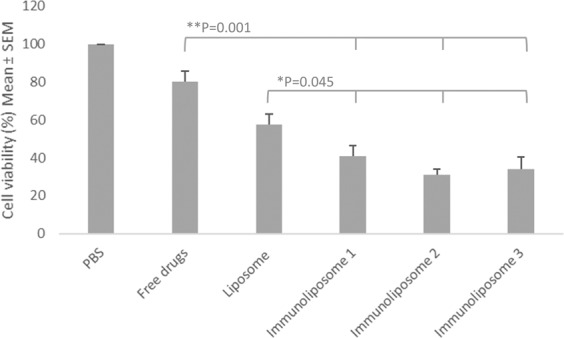
Cell viability (%) of MDA-MB-453 spheroids 14 days after seeding^a^. ^a^Values are mean of three or more experiments.

Empty liposomes and trastuzumab alone did not show cytotoxic effect on MDA-MB-453 (data not shown)^26^. Spheroid monitoring is summarized in Fig. 6. At day 14, cell viability was 80 ± 17, 57 ± 16, 41 ± 16, 31 ± 9 and 34 ± 19 when treated with free drugs, liposomes, Immunoliposome-1, Immunoliposome-2 and Immunoliposome-3, respectively (Fig. 7). Thus, immunoliposomes performed better than free drugs (p = 0.001, *t*-test) and liposomes (p = 0.045, *t*-test). However, no statistical difference was observed between the three immunoliposome batches (p > 0.05, One-way ANOVA).

## Discussion

In the present study for the first time we used QFCM approach for characterizing third generation of drug-loaded liposomes, a.k.a. immunoliposomes. Here, we worked on prototyping docetaxel-trastuzumab immunoliposome which previously demonstrated *in vitro*^7^ and *in vivo*^26^ anti-tumor efficacy. However, two major hurdles had to be overcome: first, QFCM had never been applied yet on submicrometer-sized particles and second, FCM analysis of nanoparticles was still a major challenge^27^.

Schematically, two main QFCM strategies are still in use for absolute quantification of molecules on individual particles, using either direct (e.g., Quantibrite, BD Biosciences, San Jose, CA, USA) or indirect immuno-fluorescence (i.e., QIFIkit, Dako, Glostrup, D and CellQuant Calibrator, BioCytex, Marseille, France)^13,16,17^. A third new, more specific and possibly more robust approach, is to create calibrator beads directly coated with the antigen of interest in various known amounts^28^. Such calibrator beads can be tailored to the appropriate size and antigen density range to mimic the particles of interest in the effective staining protocol. This was our present strategy, with kappa light chains of human IgG (so-called “IgHk”) as the target antigen mimicking trastuzumab molecules coated on immunoliposomes. Using transitional calibration systems, we progressively reduced beads size and antigen density from 10 µm to 1 µm and from a few dozens to a few thousands’ molecules/bead, respectively, leading us to select a set of 5 calibrator beads, so-called “IgHk calibrator beads”, exhibiting from 36 to 14900 IgHk molecules/bead.

However, a few special technological tricks still had to be developed to achieve our final goals: first, fluorescence tagging to sort-out liposomes from non-specific events (i.e., both electronical and optical background) and second, the best sensitivity as possible in terms of fluorescent detection.

Indeed, because of their low refractive index and detection as compared to polystyrene beads of similar size range, liposomes generate very faint scatter signals. To overcome this major limitation, we followed other specialists’ suggestion to use a fluorescence-based rather than a scatter-based triggering parameter^29–31^. Thus, immunoliposomes were labeled during synthesis using a lipophilic fluorescent dye (i.e. DiD) with a different color than the reporting immunological reagents. As a result, immunoliposomes were defined as dye positive, green fluorescent particles on which human IgG should be quantified.

Then, to benefit from the highest sensitivity and allow a clear resolution, using last generation cytometer Cytoflex S (Beckman-Coulter, Villepinte France) we excited phycoerythrin (PE), one of the brightest fluorochrome available, at its optimal absorption peak (i.e. 561 nm instead of 488 nm for several benchtop instruments).

Together these two tricks allowed us to detect 65% of immunoliposome population and to consider this FCM analysis as representative of all immunoliposomes.

Thus, this QFCM method provides the effective number of IgG per particle on an absolute, rather than relative, basis and allows independent measurements along time. This may be quite useful for initial and long-term quality control of the MAb coating, as illustrated here in our 6 weeks long stability study. Associated with other absolute size measurement techniques such as DLS, it may also allow calculation of true surface density, expressed as molecules per surface unit, which may become useful for comparison of immunoliposomes with different sizes when several prototypes have to be tested.

To our knowledge, this is the first description of a FCM-based quantitative analysis of antigens/receptors on submicron particles and can be easily adapted to any other target molecule that could be biotinylated. Noteworthy, QFCM can not only provide a median value for the number of molecules per particle but can also inform about the dispersion of the immunoliposome contains, as it was illustrated in Fig. 3. Thus, both median density and homogeneity of coating comes at hands, even on very small particles such as liposomes.

Finally, this new method was used to evaluate the influence of median trastuzumab density on the cytotoxic effect of immunoliposomes. To test our hypothesis, three different batches of immunoliposomes were synthesized and exhibited a 330–690 range of coated trastuzumab per liposome, respectively. As previously published^7^, Immunoliposomes displayed a steady size but mild docetaxel leakage over time. Similarly, for Immunoliposome-1 and Immunoliposome-2, coated trastuzumab slightly decreased weekly. Oppositely, coated trastuzumab on Immunliposome-3 seemed to increase over time (i.e., +190% after 45 days) raising numerous questions. It is likely that this increase in coating was related to immunoliposome DiD staining. Indeed, DiD staining is a key point in our quantification technique since only fluorescent immunoliposomes can be accurately analyzed on Cytoflex S (Fig. 2). However, as docetaxel, DiD is a lipophilic agent and is expected to leak from liposome bilayers over time. Thus, the smaller liposomes which are considered less stable^32,33^, loose rapidly their fluorescence and become invisible within a few weeks to CMF analysis. Since smaller liposomes are expected to exhibit lower number of coated trastuzumab, this loss in DiD staining could be responsible for an analysis of larger liposomes only, after a few weeks, thus resulting in increased coated trastuzumab density. Although it appeared as a limitation of our new quantification assay when applied to stored liposomes, it could be overcome using extemporaneously-synthesized liposomes or steadier nanoparticles such as inorganic or polymeric ones.

The three batches of immunoliposomes were then tested *in vitro* on bidimensional (2D) and three-dimensional (3D) models. We found that similarly to standard MTT assays^26,34^, apoptosis studies using 2D models were not suitable to discriminate the efficacy of the various immunoliposomes as compared with free docetaxel or liposomal docetaxel. Conversely when using 3D spheroids, we found that all immunoliposomes performed better than free docetaxel and its liposomal form. Although not significant, differences between Immunoliposome batches were observed because higher antiproliferative efficacy was achieved with Immunoliposome-2 and Immunoliposome-3 as compared with Immunoliposome-1. Reducing the coating probably led to a loss in cellular uptake, thus impairing trastuzumab and docetaxel cytotoxic effects. Conversely, increasing, trastuzumab coating proved to be at best equally effective but not better, and was then nothing but a waste in trastuzumab during synthesis. Indeed, although 45% more molecules of trastuzumab were coated on Immunoliposome-3, it resulted in similar cellular uptake and antiproliferative efficacy, possibly because of steric hindrance and binding-site barrier issues^35^. Thus, Immunoliposome-2, corresponding to 480 ± 110 molecules of coated trastuzumab per liposome, could be the optimal number of trastuzumab to be coated to ensure a maximal efficacy in this breast cancer model. Interestingly, preliminary *in vivo* efficacy studies have already shown its benefit over free docetaxel + free trastuzumab and antibody drug conjugate T-DM1^26^.

Although, the present technology-oriented study needs more investigations, for instance about cell Her2 expression influence^36,37^, our data already suggest that maximal density of the targeting agent is not a major requirement for achieving maximal efficacy, thus highlighting how bio-physical parameters must be finely tuned and how critical is the need for an accurate quantitation of the targeting agent on NP’s surface. Confirmation on more sophisticated *in vitro*^38,39^ and *in vivo* models may also be of major interest to better illustrate the exquisite specificity of immunoliposome-based treatments.

### Outlook

For the first time, we have developed a sensitive FCM-based quantitative method to measure the number of coated antibodies on nanoparticles. This new quantification assay can help to characterize targeting nanoparticles as part of early prototyping steps. In addition, it can also be used as a quality control tool to control batch-to-batch variations when the optimal NP has been selected. Applied to docetaxel – trastuzumab immunoliposomes in a model of Her2+ breast cancer cell line, QFCM was successfully used to precise the optimal number of trastuzumab molecules to coat to achieve a maximal efficacy. Beyond this first application, we believe that this original method could be useful to researchers looking for a rapid, simple and precise method to quantify any monoclonal antibodies used as targeting agents when developing smart nanoparticles.

## Methods

### Drugs and chemicals

Egg yolk phosphatidylcholine (PC), phosphatidylglycerol (PG), cholesterol (Chol), 1,2-Distearoyl-*sn*-glycero-3-phosphoethanolamine (PEG) and Paclitaxel were purchased from Sigma (St Quentin-Fallavier, France). 1,2-distearoyl-*sn*-glycero-3-phosphoethanolamine-N-[maleimide(polyethylene glycol)-2000 (Mal-PEG were purchased from Coger (Paris, France). Docetaxel was purchased from VWR (Fontenay sous bois, France) and DiD fluorescent tag from Thermo Fisher (Illkirch, France). Trastuzumab (Herceptin) was kindly given by Genentech (South San Francisco, CA, USA). Anti-human IgG PE (clone L1C1) and a prototyped human IgG kappa light chains quantification kit for submicron particles (e.g., immunoliposomes) were provided by Biocytex (Marseille, France) as described below. All other reagents were of analytical grade.

### QFCM method: development of a quantification assay for human IgG on submicron particles

For QFCM analysis of submicron particles both the size and measuring range of the official calibration systems (i.e. 10 µm diameter and 3,300 to 783,000 IgG/bead for QIFIkit, DAKO, Glostrup, Denmark) are far too high for the specific needs. Thus, we had to reduce both parameters towards the more acceptable diameter of 1 µm and range of a few dozens to a few thousands’ molecules/bead. This was done in successive steps as summarized in Table 2 and detailed in the Supplemental data section^13,14,17,19,28,40–46^ depicting the generation of the prototype “µ-QIFIkit” calibrator.

**Table 2:** Successive steps involved for the calibration and application of the prototype “IgHκ calibrator beads”.

Calibrator beads	QIFIkit (commercial)	Midi-QIFIkit (prototype)	Mini-QIFIkit (prototype)	µ-QIFIkit (prototype)	“IgHκ beads” (protoype)	“IgHκ beads” (prototype)
Diameter	10 µm	3 µm	3 µm	1 µm	1 µm	1 µm
Coated analyte	Mouse IgG1	Biotin-IgG1 (mouse)	Biotin-IgG1 (mouse)	Biotin-IgG1 (mouse)	REAfinity* CD151-biotin (human IgG-k)	Trastuzumab on liposomes (human IgG-k)
Coating method	Proprietary	Magnetic SA-beads	Magnetic SA-beads	Magnetic SA-beads	Magnetic SA-beads	Covalent coupling
Calibration FCM instrument	FC500 and/or Gallios	Gallios	CytoFLEX-S	CytoFLEX-S	CytoFLEX-S	CytoFLEX-S
Staining method	QIFI (indirect IF with unlabeled mouse IgGs)	QIFI (indirect IF with unlabeled mouse IgGs)	QIFI (indirect IF with unlabeled mouse IgGs)	QIFI (indirect IF with unlabeled mouse IgGs)	QIFI (indirect IF with unlabeled mouse IgGs)	Direct IF with L1C1-PE (anti-human kappa)
Laser height at interrogation point	= or >10 µm, (depending on instrument)	10 µm	5 µm	5 µm	5 µm	5 µm
Excitation λ	488 nm	488 nm	561 nm	561 nm	561 nm	561 nm
Calibration channel	FITC	FITC	PE	PE	PE	PE
Calibration curve(s)	N.A.	Fig. S1a	Fig. S1b	Fig. S1c	Fig. 1	Fig. 2

To generate “IgH_k_ calibrator beads”, 1 µm magnetic beads coated with streptavidin (MyOne-SA Dynabeads, Dynal-InVitrogen) were decorated with increasing amounts of a human IgG kappa (IgH_k_) Mab (REAfinity* CD151-biotin, Myltenyi-Biotech France), used to mimick and calibrate trastuzumab molecules present on immunoliposomes. After an incubation of 2 min, the beads were washed using a magnet (Dynal mini-MPC), suspended in PBS-BA binding buffer (PBS-0.1% BSA-0.1% NaN3) to a concentration of 80*10^6^ µS/mL (2,5*10^5^/test) and stored at 2–8 °C. Their calibration was operated by the QIFI assay (Quantitative Immuno-Fluorescence Indirect assay) as described in the QIFIkit box insert but using the 1µm-sized µ-QIFIkit beads as reference (see Supplemental data section for µ-QIFIkit generation) after 1^st^ step saturation with the anti-human kappa light chain L1C1 Mab, intermediate washing with the magnet and fluorescent staining with a PE-conjugated anti-mouse IgG (H + L) polyclonal antibody as 2^nd^-step reagent (Jackson ImmunoResearch, d = 100). Beads covering the appropriate range of IgHk were then selected for QFCM analysis of immunoliposomes.

Since liposomes (submicron particles in general) are inappropriate to afford washing steps, a direct IF no-wash protocol was then adapted for their staining. L1C1 anti-IgHk Mab was directly conjugated to PE and purified according to BioCytex internal procedures. Titration was done in the final assay with high concentration of the highest level IgHk-expressing beads to ensure saturating conditions without excess of PE-Mab. With this Mab of rather high affinity, the saturating PE-conjugate initial concentration during staining was not more than 5 µg/mL and thus less than 2 µg/mL in the final diluted suspension during QFCM analysis.

### Immunoliposome preparation

As previously described, liposomes were synthesized using the Thin-Film method^7^. PC, Chol, PG, docetaxel and Mal-PEG were mixed in a 50:19:15:1.7:1 molar ratio. Briefly, lipids were dissolved in methanol. Lipid solution was further mixed with DiD as lipophilic membrane insertable fluorescent reporter when required for liposome-oriented FCM analysis. Methanol was then removed by rotary evaporation (Laborota 4003, Heidolph Instruments, Schwabach, Germany) at 38 °C. After 30 minutes a thin lipid film was obtained. To remove the remaining solvent, this lipid film was dried under a stream of nitrogen for two hours at room temperature. The film was then hydrated with a 5% vol/vol glucose solution and large liposomes were obtained. Reduction and homogenization in size was thus achieved by two cycles of extrusion through 100 nm and 80 nm polycarbonate pore membranes (Nucleopore, Whatman) using a LipoFast LF-50 extruder. Trastuzumab was then coated using a maleimide linker^8,10^ requiring a preliminary step of trastuzumab thiolation. Trastuzumab was first dissolved in a 0.1 M sodium phosphate buffer (PBS) pH 8.0 containing 5 mM EDTA and mixed under constant shaking, for two hours at room temperature with a Traut’s reagent solution at 1:10 molar ratio (Traut’s:trastuzumab). Thiolated trastuzumab was then directly mixed with the pegylated liposomes at 1:508 (Immunoliposome-1), 1:127 (Immunoliposome-2) and 1:16 (Immunoliposome-3) trastuzumab:Mal-PEG molar ratio. The mixture was kept under constant shaking at 4 °C overnight. Unbound trastuzumab and free docetaxel were removed using 6 000 g centrifugation on MWCO 300KDa Vivaspins (VWR,Fontenay sous bois, France) followed by size exclusion chromatography on qEV columns (IZON Science, Lyon, France).

### Size and polydispersity study

Size and polydispersity index (PDI) were measured by Dynamic light scattering (DLS). Liposomes and immunoliposomes were diluted in a PBS solution and then analyzed by a Zeta sizer Nano S (Malvern instruments, UK). Liposomal preparations were considered unimodal for a PDI < 0.2^47^.

### Docetaxel entrapment efficiency

Docetaxel concentrations were measured using a validated HPLC-UV method^48^ after liquid/liquid extraction using a C18 column (25 cm × 4.6 mm, 5 µm). The mobile phase was composed of 53% of ammonium acetate buffer (35 nM, pH 5) and 47% of acetonitrile. Samples were eluted at a constant flow rate of 1.8 ml/min with UV detection (227 nm). Data were acquired and analyzed using Chemstation software (Agilent, France). Docetaxel and paclitaxel typical retention times were respectively 11 minutes and 13.5 minutes. Docetaxel entrapment efficiency was calculated using the following formula:

$${\rm{Entrapment}}\,{\rm{efficiency}}=\frac{(mg\,DOCE\,HPLC\,measured)}{(mg\,DOCE\,present\,before\,centrifugation)}\times 100.$$

### Flow cytometric detection and quantitative analysis of trastuzumab-coated Immunoliposomes

QFCM analysis was performed on highly sensitive flow cytometer, CytoFLEX S (Beckman-Coulter, Villepinte, France) using PE-conjugated polyclonal or monoclonal antibodies and 561 nm excitation laser.

To avoid unspecific events mixing with liposomes in the FCM analysis, fluorescent triggering was involved, using a generic red fluorescent signal issued from the red laser excitation point, totally independent from the PE-MAb staining which provides orange fluorescence from the yellow laser. To apply such a generic staining, liposomes were tagged with DiD fluorescent lipophilic molecules, which encapsulated in the phospholipid bilayer of all liposomes. Consequently, the triggering parameter for their analysis on the CytoFLEX S was the red fluorescence of DiD taken from the red laser.

To determine the percentage of immunoliposome detected with flow cytometry, an absolute quantification of immunoliposomes was performed using TRPS technology (qNano, IZON, Lyon France).

### Stability studies

Stability studies were performed in PBS at 4 °C, protected from light. Immunoliposome size, PDI, docetaxel leakage and persistence of coated trastuzumab were evaluated weekly for a month, then bimonthly for up to 45 days, using differential centrifugation to separate NPs from soluble material.

### Cell lines

*In vitro* experiments were carried on HER2+ human breast cancer cell line MDA-MB-453. Cells were purchased from the American Type Culture Cell (Molsheim, France) cultured in RPMI (Thermo Fisher, Illkirch, France) supplemented with 10% FBS, 1% penicillin and 0.16% kanamycin and grown in a humidified CO2 incubator at 37 °C^26^. Cells were regularly checked for cell viability, morphology and doubling time. Cells were stably transfected with dTomato lentivirus developed and kindly provided by Pr Jacques Robert (Institut Bergonié, Bordeaux, France) and selected with blasticidin to allow fluorescence imaging^26^.

### *In vitro* efficacy studies: 2D model

To evaluate cell apoptosis, we used flow cytometry with an AnnexinV/PI kit (Sigma Aldrich,St Quentin Fallavier, France). MDA-MB-453 were seeded at a density of 7 × 10^5^ cells per well in 6-well plates. After overnight attachment, cells were exposed to free docetaxel + free trastuzumab (i.e., free drugs), docetaxel liposomes + free trastuzumab (i.e., Liposome), and immunoliposomes for 12, 36 and 72 hours. For all conditions, docetaxel concentration was 2 µM and trastuzumab concentrations were 3 nM, 4 nM and 6 nM for batches 1, 2 and 3, respectively, whether trastuzumab was free or coated. Cells were then stained with 10 µl of Annexin V FITC that targets Phosphatidylserine (PS) molecules present on the outside layer of apoptotic cell membranes and 10 µl of Propidium Iodide (PI), a fluorescent DNA intercalant molecule that stains nuclear DNA when cells undergo late apoptosis/necrosis. Cells were incubated for 20 minutes à 4 °C and then washed in 2 ml of Binding buffer (i.e., Ca2+ stabilizing buffer). Cells were then centrifuged 10 min, 900 g at 4 °C and cell pellet was resuspended in 500 µl of binding buffer prior to FCM acquisition on Gallios FCMr (Beckman Coulter, Villepinte, France). Live cells were considered as AnnexinV neg/PI neg events. Cells in early apoptosis were considered as AnnexinV+/PI−. Cells in late apoptosis were considered as AnnexinV+/PI+ and finally, necrotic cells were considered as AnnexinV−/PI+.

### *In vitro* efficacy studies: 3D model

MDA-MB-453 were seeded with 20% methylcellulose solution on U-bottom 96-well plate for 24 hours before the experiment begins. Cell density was 4000 cells/well. To evaluate treatment antiproliferative efficacy on spheroids, the following conditions were tested: free docetaxel + free trastuzumab (i.e., free drugs), liposomes + free trastuzumab (Liposome), Immunoliposome-1, Immunoliposome-2 and Immunoliposome-3. Treatments were incubated 3 days after seeding. Cells were exposed continuously to treatments for a week, then treatment was repeated, and drugs incubated until day 14. For all conditions concentration of docetaxel was 8 nM. Cell viability was determined using CellTiter-Glo (Promega, Charbonnières-les-Bains), following manufacter’s guidelines and luminescent spectrophotometric reading on PHERAstar FSX (BMG Labtech, Heathfiel, UK). Spheroids were also monitored daily using a fluorescence microscope (Nikon, Eclipse TS100), coupled to digital camera.

### Statistical analysis

Similarly to all our studies^7,26^*, in vitro* experiments were performed at least in triplicate and data were represented as mean ± standard deviation (SD) or ± standard error of the mean (SEM). Statistical analyses were performed on SigmaStat (San Jose, USA). Differences between treatments were analyzed by One-Way Anova with Multiple Comparison testing or Student’s *t*-test according to data distribution and sample size.

## Supplementary information

Supplementary Information.

## References

[1] Langer R (1998). Drug delivery and targeting. Nature.

[2] Rodallec A, Benzekry S, Lacarelle B, Ciccolini J, Fanciullino R (2018). Pharmacokinetics variability: Why nanoparticles are not just magic-bullets in oncology. Critical Reviews in Oncology/Hematology.

[3] Fanciullino R (2014). Biodistribution, Tumor Uptake and Efficacy of 5-FU-Loaded Liposomes: Why Size Matters. Pharm Res.

[4] Farokhzad OC (2006). Targeted nanoparticle-aptamer bioconjugates for cancer chemotherapy *in vivo*. Proc. Natl. Acad. Sci. USA.

[5] Gu FX (2007). Targeted nanoparticles for cancer therapy. Nano Today.

[6] Cheng J (2007). Formulation of functionalized PLGA-PEG nanoparticles for *in vivo* targeted drug delivery. Biomaterials.

[7] Rodallec A (2018). Docetaxel-trastuzumab stealth immunoliposome: development and *in vitro* proof of concept studies in breast cancer. Int J Nanomedicine.

[8] Yang T (2007). Preparation and evaluation of paclitaxel-loaded PEGylated immunoliposome. Journal of Controlled Release.

[9] Zhou Z, Badkas A, Stevenson M, Lee J-Y, Leung Y-K (2015). Herceptin conjugated PLGA-PHis-PEG pH sensitive nanoparticles for targeted and controlled drug delivery. International Journal of Pharmaceutics.

[10] Choi WI (2015). Targeted antitumor efficacy and imaging via multifunctional nano-carrier conjugated with anti-HER2 trastuzumab. Nanomedicine: Nanotechnology, Biology and Medicine.

[11] D’hautcourt, J.-L. Quantitative flow cytometric analysis of membrane antigen expression. *Curr Protoc Cytom* Chapter 6, Unit 6.12 (2002). 10.1002/0471142956.cy0612s22 18770769

[12] Poncelet P (2015). Tips and tricks for flow cytometry-based analysis and counting of microparticles. Transfus. Apher. Sci..

[13] Poncelet P, Carayon P (1985). Cytofluorometric quantification of cell-surface antigens by indirect immunofluorescence using monoclonal antibodies. J. Immunol. Methods.

[14] Poncelet P, George F, Papa S, Lanza F (1996). Quantitation of hemopoietic cell antigens in flow cytometry. Eur J Histochem.

[15] Poncelet, P. *Microbeads and flow cytometry: how and why put the ‘-metry’ in immuno-cytometry?* vol. 62 (2004). 15047491

[16] Gratama JW (1998). Flow cytometric quantitation of immunofluorescence intensity: problems and perspectives. European Working Group on Clinical Cell Analysis. Cytometry.

[17] Serke S, Lessen A, Huhn D (1998). Quantitative fluorescence flow cytometry: A comparison of the three techniques for direct and indirect immunofluorescence. Cytometry.

[18] Schwartz A, Marti GE, Poon R, Gratama JW, Fernández-Repollet E (1998). Standardizing flow cytometry: a classification system of fluorescence standards used for flow cytometry. Cytometry.

[19] Bikoue A (1996). Quantitative analysis of leukocyte membrane antigen expression: normal adult values. Cytometry.

[20] Stewart JJ (2016). Role of receptor occupancy assays by flow cytometry in drug development. Cytometry B Clin Cytom.

[21] Green CL (2016). Recommendations for the development and validation of flow cytometry-based receptor occupancy assays. Cytometry B Clin Cytom.

[22] Moulard M, Ozoux M-L (2016). How validated receptor occupancy flow cytometry assays can impact decisions and support drug development. Cytometry B Clin Cytom.

[23] Lapusan S (2012). Phase I studies of AVE9633, an anti-CD33 antibody-maytansinoid conjugate, in adult patients with relapsed/refractory acute myeloid leukemia. Invest New Drugs.

[24] Engelberts PJ (2013). A quantitative flow cytometric assay for determining binding characteristics of chimeric, humanized and human antibodies in whole blood: proof of principle with rituximab and ofatumumab. J. Immunol. Methods.

[25] Rodallec Anne, Brunel Jean-Michel, Giacometti Sarah, Maccario Helene, Correard Florian, Mas Eric, Orneto Caroline, Savina Ariel, Bouquet Fanny, Lacarelle Bruno, Ciccolini Joseph, Fanciullino Raphaelle (2018). Docetaxel-trastuzumab stealth immunoliposome: development and in vitro proof of concept studies in breast cancer. International Journal of Nanomedicine.

[26] Rodallec A (2018). From 3D spheroids to tumor bearing mice: efficacy and distribution studies of trastuzumab-docetaxel immunoliposome in breast cancer. Int J Nanomedicine.

[27] Cointe S (2017). Standardization of microparticle enumeration across different flow cytometry platforms: results of a multicenter collaborative workshop. J. Thromb. Haemost..

[28] Schuster H (2017). The immunopeptidomic landscape of ovarian carcinomas. Proc. Natl. Acad. Sci. USA.

[29] Arraud N, Gounou C, Turpin D, Brisson AR (2016). Fluorescence triggering: A general strategy for enumerating and phenotyping extracellular vesicles by flow cytometry. Cytometry A.

[30] Stoner SA (2016). High sensitivity flow cytometry of membrane vesicles. Cytometry A.

[31] Libregts SFWM, Arkesteijn GJA, Németh A, Nolte-’t Hoen ENM, Wauben MHM (2018). Flow cytometric analysis of extracellular vesicle subsets in plasma: impact of swarm by particles of non-interest. J. Thromb. Haemost..

[32] Rovira-Bru M, Thompson DH, Szleifer I (2002). Size and structure of spontaneously forming liposomes in lipid/PEG-lipid mixtures. Biophys. J..

[33] Sabın J, Prieto G, Ruso JM, Hidalgo-Álvarez R, Sarmiento F (2006). Size and stability of liposomes: A possible role of hydration and osmotic forces. Eur. Phys. J. E.

[34] Sambale F (2015). Three dimensional spheroid cell culture for nanoparticle safety testing. Journal of Biotechnology.

[35] Li Y, Kröger M, Liu WK (2014). Endocytosis of PEGylated nanoparticles accompanied by structural and free energy changes of the grafted polyethylene glycol. Biomaterials.

[36] Ho-Pun-Cheung Alexandre, Bazin Hervé, Gaborit Nadège, Larbouret Christel, Garnero Patrick, Assenat Eric, Castan Florence, Bascoul-Mollevi Caroline, Ramos Jeanne, Ychou Marc, Pèlegrin André, Mathis Gérard, Lopez-Crapez Evelyne (2012). Quantification of HER Expression and Dimerization in Patients’ Tumor Samples Using Time-Resolved Förster Resonance Energy Transfer. PLoS ONE.

[37] Gaborit N (2011). Time-resolved fluorescence resonance energy transfer (TR-FRET) to analyze the disruption of EGFR/HER2 dimers: a new method to evaluate the efficiency of targeted therapy using monoclonal antibodies. J. Biol. Chem..

[38] Costa EC, Gaspar VM, Marques JG, Coutinho P, Correia IJ (2013). Evaluation of Nanoparticle Uptake in Co-culture Cancer Models. PLOS One.

[39] Lazzari G (2018). Multicellular spheroid based on a triple co-culture: A novel 3D model to mimic pancreatic tumor complexity. Acta Biomater.

[40] Poncelet, P. *et al*. Clinical applications of quantitative immunophenotyping. In: Stewart, C. C. & Nicholson, J. K. A. eds. Immunophenotyping. New-York: Wiley-Liss, 2000: 105–32*. in 105–132 (2000).

[41] Poncelet Philippe, Lavabre-Bertrand Thierry, Carayon Pierre (1986). Quantitative Phenotypes of B Chronic Lymphocytic Leukemia B Cells Established with Monoclonal Antibodies from the B Cell Protocol. Leukocyte Typing II.

[42] Lavabre-Bertrand T (1994). Leukemia-associated changes identified by quantitative flow cytometry: I. CD10 expression. Cytometry.

[43] Olejniczak SH, Stewart CC, Donohue K, Czuczman MS (2006). A quantitative exploration of surface antigen expression in common B-cell malignancies using flow cytometry. Immunol. Invest..

[44] Battle R, Clark B (2007). Quantitative analysis of human leucocyte antigen expression during culture of Epstein-Barr virus-transformed cell lines using the dako QIFIKIT. Br. J. Biomed. Sci..

[45] Poncelet, P., Franco, C. & Ruf, W. Immunological calibration of fluorescence scales for microvesicle analysis by flow cytometry. Abst. # 209, CYTO 2017, 32nd Congress of the International Society for Advancement of Cytometry, June 10–14, Boston (MA) (2017).

[46] Poncelet P (2004). Microbeads and flow cytometry: how and why put the ‘-metry’ in immuno-cytometry?. Annales de biologie clinique..

[47] Soema PC, Willems G-J, Jiskoot W, Amorij J-P, Kersten GF (2015). Predicting the influence of liposomal lipid composition on liposome size, zeta potential and liposome-induced dendritic cell maturation using a design of experiments approach. European Journal of Pharmaceutics and Biopharmaceutics.

[48] Garg MB, Ackland SP (2000). Simple and sensitive high-performance liquid chromatography method for the determination of docetaxel in human plasma or urine. J. Chromatogr. B Biomed. Sci. Appl..

