# Supplementary material for: Prototyping Trastuzumab Docetaxel Immunoliposomes with a New FCM-Based Method to Quantify Optimal Antibody Density on Nanoparticles
Source: Sci Rep. 2020 Mar 5;10:4147. doi: 10.1038/s41598-020-60856-z (PMC7057981; doi:10.1038/s41598-020-60856-z)
Supplement: Supplementary file 1 — Supplementary Information. [file 41598_2020_60856_MOESM1_ESM.docx]

**PROTOTYPING TRASTUZUMAB DOCETAXEL IMMUNOLIPOSOMES WITH A NEW FCM-BASED METHOD TO QUANTIFY OPTIMAL ANTIBODY DENSITY ON NANOPARTICLES**

A. RODALLEC^1^, C. FRANCO^2,4^, S. ROBERT^3^, G. SICARD^1^, S. GIACOMETTI^1^, B. LACARELLE^1^, F. BOUQUET^5^, A. SAVINA^5^, R. LACROIX^4,6^, F. DIGNAT-GEORGE^3,4,6^, J. CICCOLINI^1^, P. PONCELET^2^, R. FANCIULLINO^1^*

^1^: SMARTc Unit, CRCM, Inserm UMR1068, CNRS UMR7258, Aix-Marseille University, Marseille, France

^2^: Biocytex, Marseille, France

^3^: C2VN, AMUTICYT Core facility, INSERM, INRA, Aix-Marseille University, Marseille, France

^4^: Aix-Marseille University, INSERM, INRA, C2VN UMR_S1263, UFR de Pharmacie, Marseille, France

^5^: Institut Roche, Boulogne Billancourt, France

^6^: Department of Hematology and Vascular Biology, CHU La Conception, APHM, Marseille, France^.^

**SUPPLEMENTARY DATA:**

**FCM absolute quantification Context**

Indirect IF systems, beads covered with various amounts of fluorochrome allow the calibration of arbitrary scales of fluorescence intensity (FI) in terms of fluorochrome molecules. When the Fluorochrome to Protein (F:P) ratio of the antibody is known, one can infer the number of antibody molecules bound per cell (AB/C). If, in addition, care is made to use saturating concentrations of Mab, the so-called Antibody Binding Capacity (ABC = maximal AB/C at saturation) is a measure of the number of antigen copies, under the additional (most likely) assumption of monovalent binding between the Mab and the antigen. A commercially available kit system for this direct IF approach is found within the Quantibrite (BD Biosciences, San Jose, CA, USA). 10 µm Beads coated with defined quantities of Phycoerythrin (PE) [39] offer a calibration system to relate Median Fluorescence intensities (MFI, expressed in a.u.), with numbers of PE molecules/cell. Using custom-designed PE-Mab conjugates with guaranteed F:P ratio of 1:1, the number of Antibody (molecules) Bound per Cell (Ab/C) can be gained. In saturating conditions, AB/C = ABC, the maximal amount of Mab that a cell can bind, a measure of the level of expression of the antigen of interest.

The alternative approach involving indirect IF uses non-intrinsically fluorescent beads covered with known amounts of mouse IgG to mimic cells covered with mouse Mab. Both calibrator beads and cells (or particles) are then fluorescently stained with a 2nd layer of FITC- (or PE-) labeled polyclonal anti-mouse Ig reagent. This QIFI assay was described already in 1985^9^ and was adapted by BioCytex (Marseille, France) as commercial kits (i.e., QIFIkit, Dako, Glostrup, D and CellQuant Calibrator, BioCytex, Marseille, France). Their 10 µm-sized calibrator beads, covering a range of a few thousands to 800,000 IgG molecules/particle are well suited for QFCM on cells but not on submicron particles and this adaptation to low sizes and calibration range was one prerequisite of the present study.

**FCM method: Development of a quantification assay for submicron particles**

QIFIkit (distributed by Dako, Glostrup, D) is a commercial product created by BioCytex and used for the quantification of antigens on cells^9,10,13,15,26-32^.

It consists of a cocktail of five positive bead subsets plus a blank bead, all of 10 µm in diameter and coated with different, but well-defined quantities of a mouse IgG mimicking monoclonal antibody (Mab) molecules bound on cells. The number of Mab molecules on the six bead populations ranges from 0 (“blank bead”) to ~800,000, depending on the precise lot number.

QIFIkit beads and cell-optimized calibrators in general are facing two separate problems for quantification of antigens on submicrometer-sized particles in general, including e.g. extra-cellular vesicles (EVs), coated latex particles for immune-assays and immunoliposomes as considered in this study: first, diameter compatibility and second, precision for low levels of antigens.

First, calibrator beads should ideally feature a size of the same order of magnitude as the particles they are used to mimic and calibrate. In addition, they should not be bigger than the laser spot size (height) used to excite the bound fluorescent reagents so that each bead can be totally enlighted when being in the middle of the beam. Whereas conventional flow cytometers, such as e.g. Beckman –Coulter Gallios, have laser spot size of 10 µm or more and are thus most appropriate for calibration with 10 µm QIFIkit beads, recent generation instruments such as CytoFLEX, more adapted to submicron particle analysis, generally use smaller spot size, here 5 µm height.

Second, the calibrator beads should cover a range of values encompassing the levels to be measured on the particles of interest, in the present case a few dozens of molecules up-to a maximum of a few thousands. Thus, smaller beads with much lower auto-fluorescence and high homogeneity of both size and coating must be used for measuring low levels of ligands on submicrometer-sized particles.

To solve these problems, the official QIFIkit tool was adapted to render it compatible with specifications of our nanoparticles by miniaturizing it in successive steps [33].

First, high levels of biotinylated mouse IgG (SBTF1, CD142 IgG1 from Biocytex) were coated on approximately 3µm streptavidin magnetic beads (M280 Dynabeads, Dynal/InVitrogen). We used QIFIkit, our current QFCM tool including 5 positive bead subsets ( 3,300-13,000- 41,000- 126,000 and 783,000 IgG/bead in this batch) plus a blank bead, all of 10 µm in diameter, and a polyclonal goat anti-mouse Immunoglobulins (H+L)-FITC coupled to quantify the number of murine IgG on the smaller 3µm-sized so-called “midi-QIFIkit” beads thus covering a range of 20,000-150,000 IgG/beads (see Supp Fig. 1a). These Data were generated on a Gallios flow cytometer (Beckman-Coulter, Villepinte France) using the blue laser-excited fluorescence of FITC.

Second, these 3 µm “midi-QIFIkit” beads were used to scale-down to a lower range the QIFI assay into CytoFLEX (Beckman-Coulter, Villepinte France), a flow cytometer equipped with a 5 µm high illumination spot. Then, a second set of the same 3 µm beads was generated with lower amounts of biotinylated mouse IgG/bead and quantified versus the high-level “midi-QIFIkit” beads on CytoFLEX S. To improve the sensitivity of fluorescence detection, the reporter secondary reagent was switched from FITC (blue 488 nm laser excitation) to PE (for yellow 561nm laser excitation) using a new polyclonal goat anti-mouse Immunoglobulins-PE (Jackson Immunoresearch). The linear relationship among the high levels (HL) of midi-QIFIkit 3 µm beads now used as reference served to calibrate the lower levels (LL) of mini-QIFIkit, providing beads with a range of 36 to 14,900. Correlation of MFI versus predicted values of each bead gave r^2^ >0.95, see Suppl Fig. 1b).

Third, the same approach was applied to coat and calibrate 1 µm mouse IgG beads. A few 3 µm beads, chosen from both midi- and mini-QIFIkit series and covering a range of 2,000 to 150,000 IgG/µS were used to quantify the 1 µm µ-QIFIkit beads on CytoFLEX-S in the PE channel (see Suppl Fig. 1c). These calibration experiments showed a good linearity among 3 µm beads (MFI versus IgG/µS values of each bead: r^2^ >0.95) and, by extrapolation, provided a series of individual 1 µm “µ-QIFIkit” beads covering a range of 20 - 14,500 IgG/µS.

**FCM method: Development of a quantification assay for submicron particles**


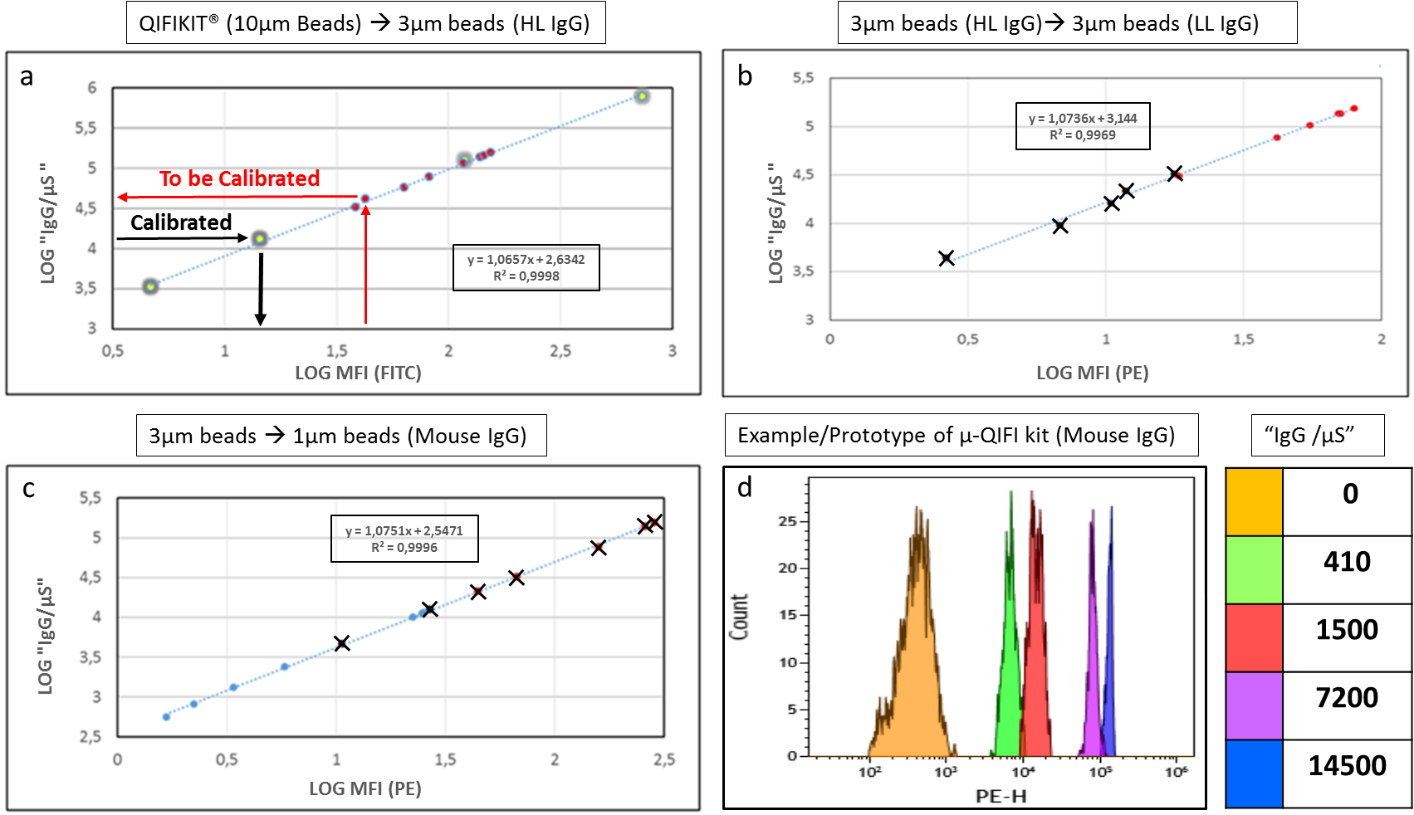


Supp. Figure 1: From QIFIkit to µ-QIFIkit

(A) Quantification of mouse IgG on 3 µm “midi-QIFIkit” beads (
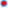
) using 10 µm QIFIkit beads as reference and FITC-conjugated anti-mouse IgG reagent as fluorescent reporter on Gallios instrument (
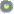
). The linear relationship linking Log (MFI) to the amount of IgG/bead (µS) is used to calculate the IgG/bead values for the new beads: Log(IgG/µS) = 1.0657x Log(MFI) + 2.6342, r² = 0.9998 and IgG/µS = 10 ^Log(IgG/µS)^

(B) Scale-down of “midi-QIFIkit” (
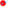
) to the lower IgG levels (
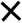
) of “mini-QIFIkit” using PE-conjugated anti-mouse IgG reagent on CytoFLEX-S . Same calculation as in a) with Log(IgG/µS) = 1.0736x Log(MFI) + 3.144, r² = 0.9969

(C) Quantification of mouse IgG on 1 µm “µ-QIFIkit” beads (
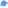
) using 3 µm-sized “mini-QIFIkit” beads as reference (
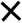
) and PE-conjugated anti-mouse IgG reagent as reporter on CytoFLEX-S. Log(IgG/µS) = 1.0751x Log(MFI) + 2.5471, r² = 0.9996

(D) Example of PE fluorescence profiles of mouse IgG detected on 1 µm “µ-QIFIkit” beads

**QNano: Measurement of liposome concentration**

As previously published by other teams^49^, TRPS technologies are of high interest to determine nanoparticle concentration. To measure our liposome concentration in our samples we then used qNano (IZON, France) with an 85-500 nm nanopore (NP200, IZON, France) and samples were diluted in PBS to be in a range of 1.10^6^ to 1.10^9^ particles per ml before 10µl analysis. Considering this technique sensitivity (< 50 nm), our homogenous liposome population (i.e., PDI < 0.2) and size (i.e., 140 nm) we considered that all liposomes were counted and this concentration was used as reference to evaluate the percentage of liposome detected with FCM (Supp. table 1).

**Supp. Table 1:** Summary of liposome concentration (nanoparticle/ml) measured or detected using qNano or FCM in our samples that were pure or diluted up to 1/8 in PBS.

|  | No dilution | Dilution 1/2 | Dilution 1/4 | Dilution 1/8 |
| --- | --- | --- | --- | --- |
| qNano | 4.38.10^7^ | 1.76.10^7^ | 5.14.10^6^ | 3.35.10^6^ |
| FCM | 1.910^7^ | 8.4.10^6^ | 5.5.10^6^ | 2.2.10^6^ |

***In vitro* efficacy studies: 2D model**


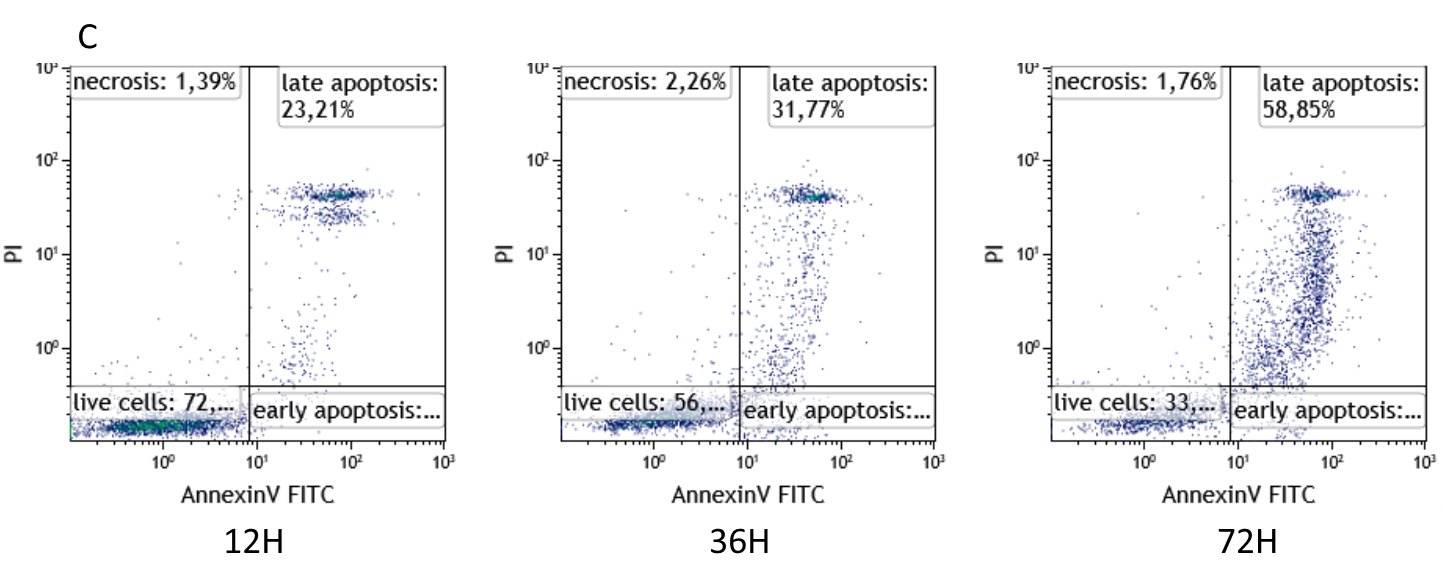


**Supp. Figure 2:** Apoptosis evaluation: representative dot plots of FCM analysis on MDA-MB-453 cells. Assays were realized 12H, 36H and 72H after treatment induction and both cell impermeant dye (PI) incorporation in dying/dead cells and Annexin V binding on apoptotic cells were monitored.

**Supp. Figure 3:** Monitoring of cell population (%) in early (A, C and E) and late (B, D and F) apoptosis when treated with three batches of free drugs (A and B), liposomes (C and D) or immunoliposomes (E and F) for 12, 36 and 72 hours^a^


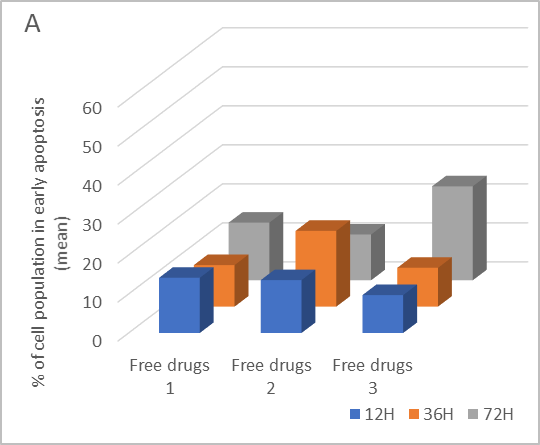

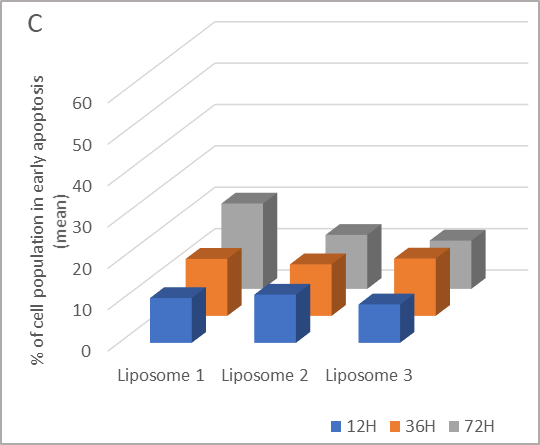

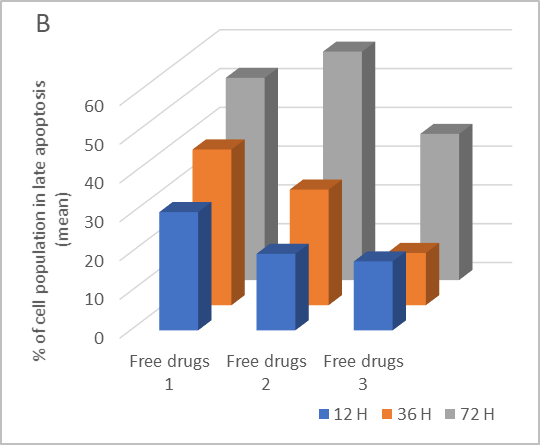

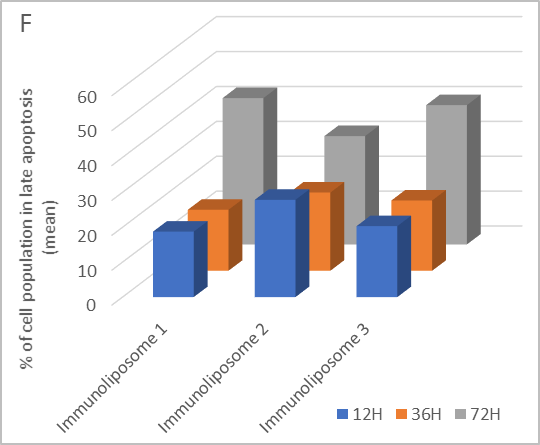

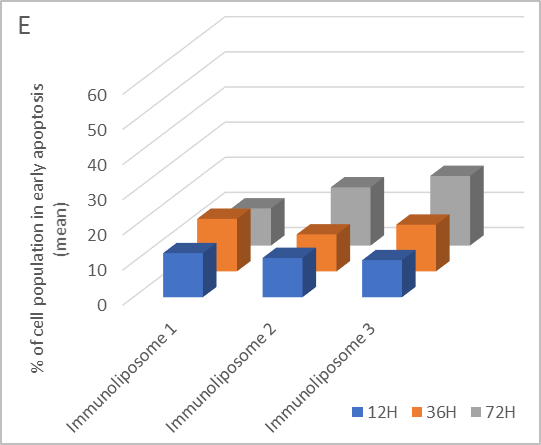

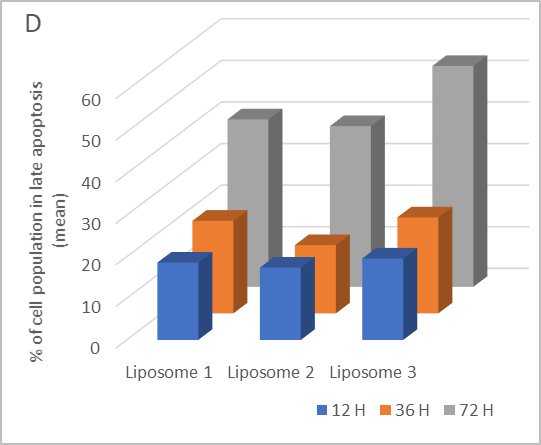


^a^: values are mean of three or more experiments
